# The Neophobia Hypothesis: nest decoration in birds may reduce predation by corvids

**DOI:** 10.1098/rsos.250427

**Published:** 2025-04-16

**Authors:** Magne Husby, Tore Slagsvold

**Affiliations:** ^1^ Section of Science, Nord Universitetet, Levanger, Norway; ^2^ Department of Biology, University of Oslo, Oslo, Norway

**Keywords:** antropogenic material, corvids, fear of feathers, nest decoration, nest material, nest predation

## Abstract

Many birds suffer heavily from nest predation, selecting several behaviours to avoid the risk. Corvids are among the most serious nest predators. However, they are also among the most neophobic of any birds. We suggest that nesting birds may take advantage of this fear by decorating the nest with anthropogenic materials that are novel to the predators (termed the Neophobia Hypothesis). They may also add large, conspicuous feathers that may indicate a site where a bird has recently been killed. In a study in the field, we found that territorial Eurasian magpies *Pica pica* waited for a longer period to remove eggs from artificial nests decorated with a shiny metal teaspoon, or with large, white feathers compared to adjacent artificial control nests with no decoration. On a landfill, where the birds had become more habituated to forage among anthropogenic material, common ravens *Corvus corax* also avoided nests decorated with a teaspoon or with feathers. The study supports the hypothesis that birds may suffer less nest predation by corvids if they decorate the nest with anthropogenic material or with large feathers.

## 1. Introduction

Some birds are attracted to conspicuous and shiny objects and may bring these items to the nest, such as large feathers and pieces of plastic [1–3]. For instance, the Eurasian magpie *Pica pica* (termed magpie below) has the reputation of stealing bright objects such as jewellery, and in Britain, the species has been used by the police to symbolize the thief [4]. There is little evidence for the reputation but in a few cases, shiny objects have been found in magpie nests [5]. Recent reviews have shown that anthropogenic materials are present in the nests of many bird species [3]. For instance, black kites *Milvus migrans* and red kites *Milvus milvus* may decorate the nest with white plastic [2,6], and corvids may use scraps of netting, telephone and electric wires and even anti-bird spikes removed from buildings in their nest [7]. The satin bowerbird *Ptilonorhynchus violaceus* may add conspicuous objects to the bower [8], and the burrowing owl *Athene cunicularia* may arrange all sorts of anthropogenic material at the entrance of its burrow in addition to faeces of animals [9]. By decoration, we mean the use of material that does not seem to function for insulation or structural support.

The reason why birds decorate their nest with anthropogenic material remains poorly understood [3,10], although some patterns have been found, such as relationships between the use of anthropogenic material and nest type and sexual dimorphism of the species [11]. Costs have also been identified because the material may reduce insulative properties and subsequently cause injury and mortality of the chicks and the adults, such as problems with ingestion and entanglement in thread-like plastic [3,11–13]. A parsimonious explanation is that anthropogenic material is often easy to discover and that it has become more common, in particular in urban areas [9,10,14]. The objects may serve as structural support although anthropogenic material may be chosen also when natural objects are present [15].

An alternative explanation is that ornamentation of the nest signals high quality of the builder [11] which may increase mating success [16], increase parental investment by the mate [17] and reduce the risk of cuckoldry [18]. The decoration may also send a signal of ownership and high social rank to conspecific intruders [2,19–21]. The behaviour may be costly due to increased detection of the nest by rivals and predators and to costs of collecting the material, and thus serve as an honest signal of status because it is individuals that are in good condition [17], and in their best age of life with regard to reproduction [2] that decorate the most. Thus, use of anthropogenic material and feathers may be considered an extended phenotype [22,23]. However, nest decoration may also increase the detectability of the nest to enemies, as shown by experiments [24,25].

Here, we suggest a new explanation for why birds decorate the nest with anthropogenic material, termed the Neophobia Hypothesis. Neophobia is defined as the degree of aversion towards objects or situations that are evolutionarily novel, or the avoidance of an environmental element that has not been experienced previously [26]. Neophobia is considered to be an adaptive response to conditions of high risk and uncertainty and may thus play a major role in animal ecology [27]. We suggest that birds decorate their nest with novel objects to trigger a fear response in their enemies, thereby reducing the risk of nest predation, egg dumping and nest usurpation. Because of neophobia, it may take some time to become familiar with an object. However, when an individual has overcome the fear, it may bring the item to the nest where it subsequently may trigger neophobic responses in intruders. In many bird species, the nest is particularly vulnerable during egg laying because the female must leave the nest site for foraging. Birds typically decorate their nest before and during the egg-laying period but less so after incubation has begun [2,28].

**Figure 1.**
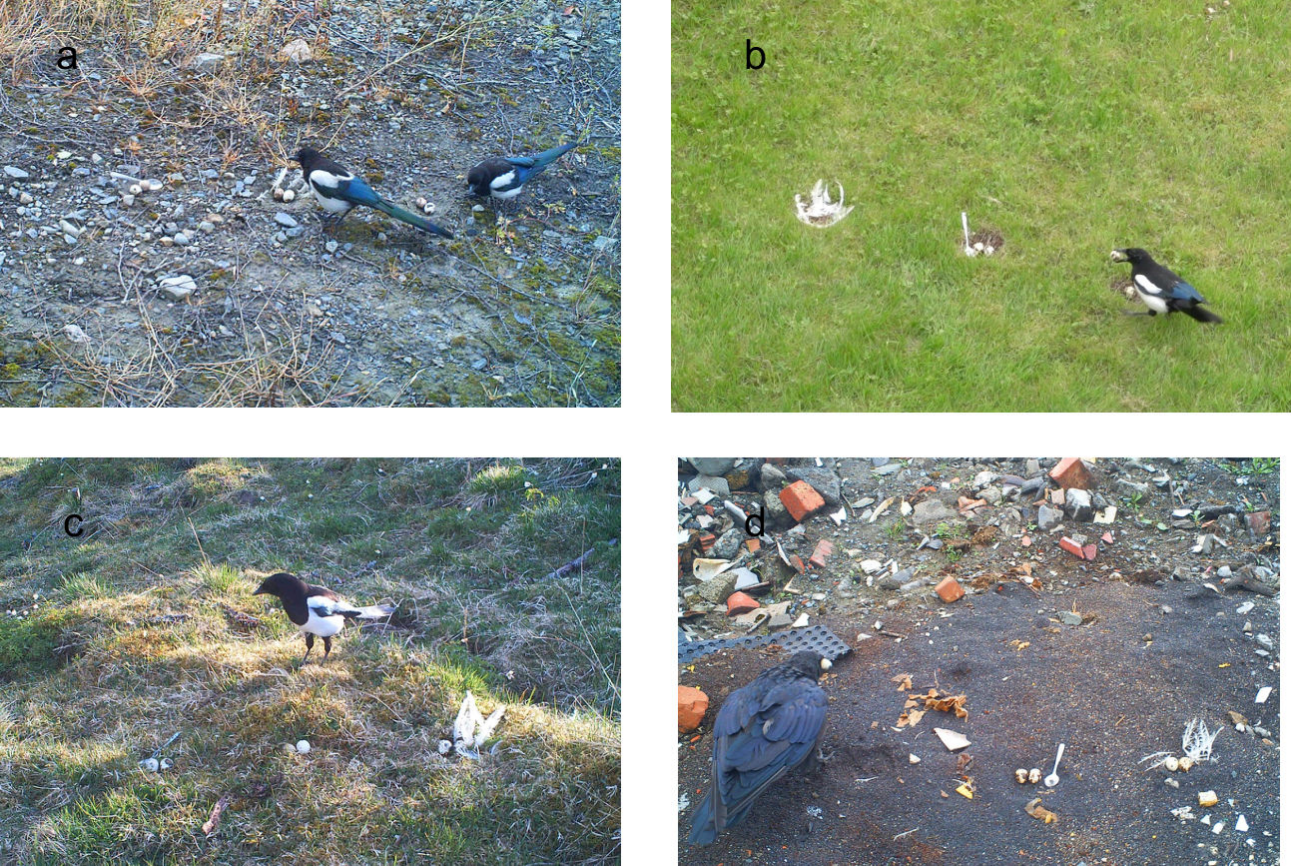
Three artificial nests were placed in open habitats on the ground within a meter to ensure that they were easily detected simultaneously by the focal predator; (a–c) magpies in their own territory and (d) a raven at a landfill. The nests were presented with quail eggs and decorated with white hen feathers, a teaspoon or nothing (control). In this example, both a magpie (b) and a raven (d) grasped an egg in their beak from the control nest.

We tested the Neophobia Hypothesis by studying depredation of eggs in artificial nests containing a metal teaspoon (figure 1). We focused on two generalist nest predators, the magpie and the common raven *Corvus corax* (termed raven below). The magpie was chosen because the species is common in our study area and because the birds and their large nests are easy to locate and observe. We studied ravens because of their large numbers occurring at a local landfill. Corvids, such as jays, magpies, crows and ravens, are among the most serious predators on bird nests in general [24,29–31] and also in our study area [32]. At the same time, these birds are among the most neophobic species of any bird [5,33] which is typical for innovative animals [34,35]. Animals may show neophobic responses to novel areas, objects, odours and/or sounds [27]. For instance, an experimental study showed that magpies hesitated to feed at sites where novel metal items were placed in a pile close to the food [5], and five species of corvids were more neophobic than seven non-corvid species towards a number of object types that varied in novelty when presented on feeding tables across rural and urban habitats [33]. We asked whether decorating the nest with conspicuous anthropogenic material would cause aversion responses in the corvids.

The experiments also allowed a study of another hypothesis, namely, the Fear of Feathers Hypothesis [36]. The hypothesis is based on the observation that many cavity-nesting birds, such as blue tits *Cyanistes caeruleus*, and tree swallows *Tachycineta bicolor*, add some large feathers on the top of their nest [17,19,37]. This may cause prospecting birds to avoid entering the nest cavity after only looking at the nest while standing in the cavity entrance and thus avoid usurpation and egg dumping [36]. The reluctance seems to be caused by a fear of predation rather than respect for the nest owners [38]. We asked whether the presence of a few reflective feathers also elicits a fear response to enter a nest in corvids. Birds are familiar with encountering feathers of other birds on the ground, e.g. left from moulting birds. However, a pile of feathers may be associated with a recent killing of a bird by an avian or a mammalian predator and thus with a site that should be avoided. The fear should also persist at landfills because such places are attractive not only to foraging corvids but also to their predators, including foxes, mustelids and birds of prey. Thus, in the present study, we expected both magpies and ravens to avoid nests decorated with feathers irrespective of habitat.

We presented three types of artificial nests on the ground at each trial site in the field, each with quail *Coturnix* spp. eggs, adding either (1) a metal teaspoon, (2) large, white hen feathers or (3) nothing to the nests (control; figure 1). We used wildlife cameras to observe egg predation by birds and mammals, asking whether magpies were less likely to feed on eggs from some of the nests that we provided on their territories. The primary objective of the experiment at the landfill was to study nest predation by ravens. However, we also report a few cases of depredation by mammals, mainly by the red fox *Vulpes vulpes*. We expected no fear of the hen feathers in the mammals which feed not only on eggs and nestlings but also on adult birds and thus are familiar with feathers associated with food. We also expected low levels of fear to the nests with a teaspoon because mammals seem in general to be less neophobic than birds [26]. Animals of higher trophic position also have less reason to show predator neophobia [26].

## 2. Methods

### 2.1. Study area and study species

The study sites were located in the western part of the mid-boreal forest zone in Norway, in Frosta, Levanger and Verdal communities (about 63°37−44′N, 10°37′−11°33′E). The study of magpies was conducted in gardens in farmland. In northern Europe, magpies defend all-purpose territories with a size of about 5−7 ha [4,39]. The species is sedentary [40] and adult survival rate is high. Thus, in our study, the focal birds probably had detailed experience within their territory. In the study area, the magpies start egg laying in April and May and care for the offspring until at least August [41,42]. The mean distance between the magpie trial sites was 351 m (s.d. = 158, range 77−880 m, *n* = 68). The separation of territories was based on observations of birds and nests.

The study of ravens was at a large landfill (Skjørdalen Avfallsdeponi, Innherred Renovasjon; diameter 470 m) where sometimes more than one hundred corvids, mostly ravens, were searching for food. The trials were conducted in the breeding season and thus presumably when most of the older territorial ravens were present in their nesting area. Therefore, most ravens were probably non-breeding yearlings and 2 year-old birds because ravens do not breed until they are (2)−3 years old [39]; in accordance with the birds that we could age from the photos, yearling ravens having a browner plumage colour than older birds [39]. During the present study, pieces of plastic and metal, including spoons, were common at the landfill. Food was not abundant but a building with organic waste was accessed by a few birds simultaneously because of some holes in the walls. Predators observed at the landfill were stoats *Mustela erminea,* pine martens *Martes martes*, red foxes, goshawks *Accipiter gentilis*, peregrine falcons *Falco peregrinus* and white-tailed eagles *Haliaeetus albicilla*.

### 2.2. Artificial nest experiment

The artificial nests were made by a depression on the ground simulating nests of species of waders (Charadrii and Scolopacidae), hazel grouse *Tetrastes bonasia* and other ground-nesting birds in the study area. In most magpie territories, we used moss or other vegetation in the artificial nests found at the local site (figure 1a–c). The nests constructed at the landfill were all on bare ground (figure 1d) without any lining and thus appeared similar to the surrounding ground. All three nests at a focal site were identically built and placed on a line within a metre to ensure simultaneous detection by the predator(s). After the three nests of a trial had been made, the same number of quail eggs were added to each. Then it was decided by throwing a dice which nest should have (a) nothing else (control), (b) 10−15 white feathers collected at a local chicken farm and about 3−13 cm long or (c) a shiny, metal teaspoon of 16 g and 13 cm long.

To increase the probability of predator detection, most nests contained two eggs. In 2022, we used one quail egg and one plasticine egg in each nest of six magpie trials. In 2023 and 2024, we used one or two quail eggs. Because of temporary shortage of quail eggs, only one quail egg was laid in each nest in 11 trials with magpies and in 22 trials with ravens. The nests were probably easier to discover by the birds from the presence of a shiny metal teaspoon and the white feathers than from the content of one or two eggs. The quail eggs provided were on average 33.6 mm ± 2.0 s.d. long and 26.5 mm ± 0.8 s.d. wide (*n* = 34). Such eggs are found to be acceptable for studies of nest predation rates in the wild [43,44] because of their size and the size of the main nest predators in the respective study areas [45]. In the present study, the magpies and ravens were able to grasp a quail egg in the beak (figure 1b,d).

The trials always started in daylight (between 8.20 a.m. and 9 p.m. of the day) and in most cases they lasted until all eggs had been removed (after a maximum of 23 days). In two cases, a trial was terminated before all eggs had been removed but after the predation order of the three focal nest types was known. Nest predation was recorded by wildlife cameras (Wingcam II TL; fixed on highest resolution, 8 MP) that saved the photos in jpeg format. We ensured that all the nests of a trial were within the field of view. The camera was placed in a neighbouring tree or on a 1.5 m high metal pole about 2 m away from the nests. It took a photo every minute independent of presence of predators.

We conducted only a single trial on each territory of the magpies and used different territories in different years. Therefore, we assumed that different birds were occupying the different sites based on their spatial distribution. The 78 accepted trials were conducted in July 2022 (*n* = 6), in April–May 2023 (*n* = 24) and in April–June 2024 (*n* = 48).

The 60 accepted trials for ravens were conducted at the landfill in June–July 2023 (*n* = 15) and April–May 2024 (*n* = 45). At the landfill, we used different sites each time with a distance to the closest accepted nest site of 5−40 m in 2023 and 20−130 m in 2024. The sites were chosen based on guidance and restrictions from the local authorities responsible for the landfill, and thus dependent on roads for trucks, construction machines, etc. In 2023, we used 4−6 trials per day, and in 2024, we used 2−9 trials per day. The change of design was to minimize the potential for habituation. Sometimes more than one hundred ravens were present at the landfill and often more than one bird was seen close to the artificial nests in a photo. We assumed that different birds removed the first egg of a trial and thus that the trials were independent.

In all accepted trials, one or more individuals of the same predator species were observed one or more times. If a nest was not depredated, the predator was not observed, or it belonged to another species than a magpie on its territory or a raven or a mammal at the landfill, the trial was excluded. For the mammals, we used the data for the pine martin (*n* = 1) and the red fox at the landfill (*n* = 14). However, the results for the mammals should be treated with care because the same individual red fox may have been responsible for the predation at several trial sites. The ravens only depredated nests on top/central parts of the landfill, whereas predation by a mammal was always at the outskirts. On the magpie territories, only one setup was depredated by a red fox. This was too few to be included in the analyses.

### 2.3. Statistics

We analysed the photos by carefully watching on a computer screen every photo recorded during the periods when eggs were removed, to identify the nest predators and to record the order of which nest was depredated and the hour of day of egg removal. From the photos, it was possible to see that an egg had disappeared from a focal nest even if the predator was not observed. If two nests were depredated between two successive photos, they were given the same predation order and time. This occurred five times in the case of the first egg for magpie trials and nine times for raven trials. In these cases, when the frequency for a nest to be depredated first was calculated, we assigned a value of 0.5 for each of the two nests. The order for the second nest type to be depredated was shared between two nests in the case of three magpie trials and seven raven trials. If a predator was observed removing an egg or was observed within a couple of metres from a nest during a trial (figure 1), we assumed that it was a member of the same species that had removed the other eggs also when no predator was directly observed on all relevant photos.

The values for the time elapsing from the start of a trial and to depredation of the first egg did not follow a normal distribution, nor after log transformation (Shapiro–Wilk test). Thus, we used non-parametric tests (Mann–Whitney *U*-test, Wilcoxon paired two-sample test, Spearman rank correlation). We used chi-square tests to compare frequencies. SPSS v. 28 was used and two-tailed tests with a significance level of 0.05.

## 3. Results

### 3.1. First egg removed by magpies and ravens

**Figure 2.**
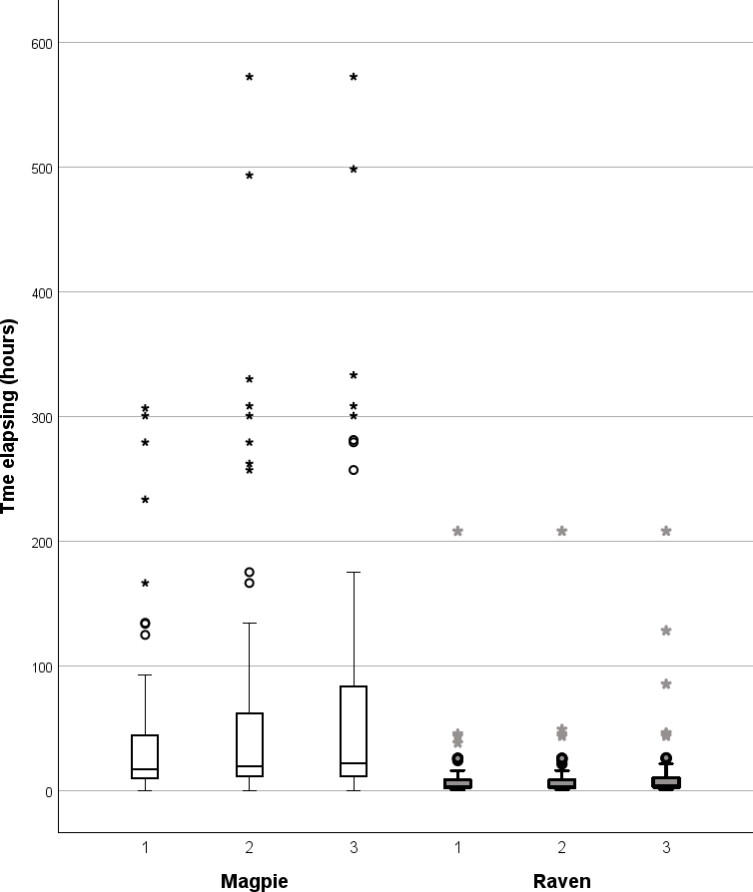
Nest predation by magpies on their territories (left, *n* = 78) and by ravens at a landfill (right, *n* = 60). At each trial site, three artificial nests were provided each with quail eggs (figure 1). Shown is a box-plot of the time (hours) elapsing from the start of a trial and the time the first egg was removed by the predator from the first, second and third depredated nests. The box-plot shows the first and the third quartile and the median. The maximum and minimum values are shown in case the data do not extend to the ends of the whiskers. Values outside the whiskers are shown as mild (^o^) or extreme (*) outliers.

The first egg was removed much later by magpies from the nests offered on their territories than by ravens at the landfill (figure 2; magpie: median 16.5 h, range 6 min–12.8 days, *n* = 78; raven: median 2.8 h, range 28 min–8.7 days, *n* = 60; Mann–Whitney *U*-test, *n*_1_ = 78, *n*_2_ = 60, *z* = − 5.30, *p* < 0.001). Some photos and observations from distance showed that a nest predator could stay in the vicinity watching the nests but hesitating to approach.

**Figure 3.**
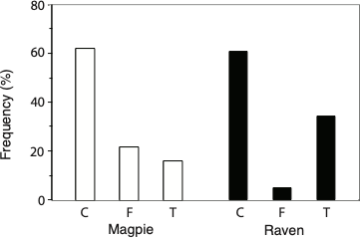
The percentage of trials when a nest with quail eggs was the first to be depredated by magpies in their territories (left, *n* = 78) and by ravens at a landfill (right, *n* = 60). At each trial site, three artificial nests were provided, either not decorated (control, C), decorated with white hen feathers (F) or with a shiny teaspoon (T; figure 1).

In both species, the distribution of which type of nest that first was depredated differed significantly from random choice (magpie: *χ*^2^_2_ = 29.6, *p* < 0.001; raven: *χ*^2^_2_ = 28.1, *p* < 0.001). The distribution also differed between the two species (2 × 3 contingency table, *χ*^2^_2_ = 10.54, *p* = 0.005). In about 60% of the trials in both species, the first egg was taken from a control nest (figure 3). For instance, during the first 2 h of a trial, eight pairs (10%, *n* = 78) of magpies had started the depredation and all took an egg from a control nest first. During the first 2 h, 14 ravens (23%, *n* = 60) had started nest predation and 68% took the first egg from a control nest. Ravens were less likely than magpies to depredate a nest with feathers first (in only 5% of the trials; in 22% of the trials for magpies), whereas magpies were less likely to start depredation of the nest with a teaspoon than the ravens (16% versus 34%, figure 3).

### 3.2. Second egg removed by magpies and ravens

**Figure 4.**
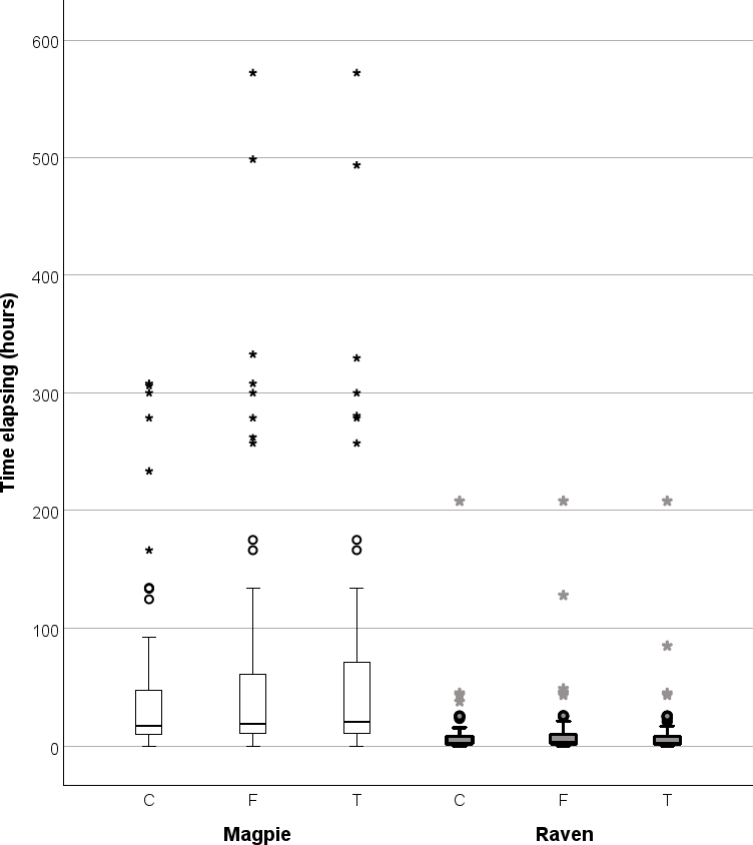
Nest predation by magpies on their territories (left, *n* = 78) and by ravens at a landfill (right, *n* = 60). At each trial site, three artificial nests were provided each with quail eggs. Shown is a box-plot of the time (hours) elapsing from the start of a trial and the time the first egg was removed by the predator from the control nest (C), the nest decorated with white hen feathers (F) and the nest decorated with a shiny teaspoon (T; figure 1). For explanation of box-plots, see figure 2.

In one case, the magpies spent more than 22 days from when the control nest was depredated until one of the other nests was depredated despite the short distance between the nests. In another trial, it took more than 18 days before the same happened. Separate pairwise comparison for the time of onset of depredation of the nest with feathers versus the control nest showed significant differences in both species (figure 4; Wilcoxon paired two-sample test; magpie: *z* = −3.95, *n* = 78, *p* < 0.001; raven: *z* = -5.57, *n* = 60, *p* < 0.001). For the nest with a teaspoon versus the control nest, a significant difference also existed in both species (same test; magpie: *z* = -5.76, *n* = 78, *p* < 0.001; raven: *z* = -2.48, *n* = 60, *p* = 0.013).

Per definition, the second nest was always depredated after the first, causing a positive correlation between the times of occurrence of the two events (Spearman rank correlation; magpies: *r_s_ =* 0.90, *n* = 78, *p* < 0.001; ravens: *r_s_ =* 0.95, *n* = 60, *p* < 0.001). In general, the birds depredated the nests in rapid succession, with a similar time interval between the onset of predation of the first and the second nest in the two species (magpie: median 2 min, range 0 min–22 days; raven: median 2 min, range 0 min–11 h; Mann–Whitney *U*-test between the two species, *n*_1_ = 78, *n*_2_ = 60, *z* = −1.41, *p* = 0.16). There was no difference between the species in the interval between the onset of predation of the second or the third nest (same test and trials, *z* = −0.70, *p* = 0.48).

### 3.3. First egg removed by mammals

In 15 trials around the landfill, the nest predator was a pine marten or a red fox. The mammals removed the first egg from a control nest in three of the trials, from a nest with feathers in four trials, and from a nest with a teaspoon in eight trials. The distribution differed significantly from those of the two corvids (magpie: *χ*^2^_2_ = 11.58, *p* = 0.003; raven: *χ*^2^_2_ = 10.19, *p* = 0.005).

## 4. Discussion

### 4.1. The Neophobia Hypothesis

In the present study, almost all nests were depredated eventually. The risk of depredation was thus equal among treatments. However, shorter latency to depredate control nests suggests that the risk might be lower for decorated nests, depending on duration of exposure. The study provides support for the Neophobia Hypothesis because both corvids were less likely to remove an egg from a nest decorated with a novel object, namely, a shiny teaspoon. However, the effect was less in the case of the raven probably because at the landfill the birds were already familiar from searching for food intermingled with shiny anthropogenic material in various sizes, including teaspoons. Mammalian predators are thought to be less neophobic than corvids [26], and, in our study, they did not hesitate to remove eggs from any of the decorated nests at the landfill compared to the control nests.

The results for the two corvids were from two independent experiments. The experiments were not directly comparable because they were conducted in different habitats and the magpies were territorial and probably older and more experienced than the ravens. The trials were conducted in the breeding season when most of the older ravens are present in their respective nesting area. Probably, most of the ravens at the landfill were only 1−2 years old. Low rank and lower ability of obtaining food for younger than older birds may cause them to be less neophobic [26]. During the experiment at the landfill, food was steadily provided although in small amounts. The many birds present may have increased competition for the eggs and selected for quick decisions during foraging, in contrast to the foraging of a co-operative pair of birds on their own, familiar territory. In addition, at the landfill there were usually many birds present that could detect and warn about approaching predators, causing a lower chance for each individual forager to be attacked. At a landfill, birds and mammals may stay for several days and become familiar with anthropogenic material. Birds, including corvids, may habituate to novel items over time [5], causing for instance neophobia to be less in urban than in rural areas [33].

We cannot exclude the possibility that a few ravens were involved in more than one trial, possibly causing habituation. However, our findings would be conservative regarding the present hypotheses predicting that novel items in the nests would cause fear. Ravens are long-lived and nesting birds exhibit strong site fidelity and occupy the same home-range areas across years. They prey on both eggs and nestlings of other birds and such food may be of great importance for their breeding success [46]. It would be interesting to study whether ravens are less likely to depredate nests decorated with anthropogenic material when the ravens are older and nesting than when they are younger and foraging at a landfill.

On the magpie territories, the median time elapsing until the first egg was removed from a nest was as much as 17 h, with a maximum of 13 days. This occurred although the nests were easily detected by the resident birds in an area familiar to them (figure 1). Actually, magpies were seen watching the artificial nests in a tree from a distance a long time before the birds had started to remove eggs. Probably, the behaviour of the magpies was not caused by the species being more wary in our population than elsewhere because magpies may be less wary in Norway than for instance in England caused by less persecution in the past [4].

The magpies showed a strong neophobic response to the nest decorated with a teaspoon. This supports the findings of a field study of magpies in England where metal screws (7 cm long, four at each site), foil rings or pieces of aluminium foil were placed on the ground next to monkey nuts [5]. The magpies were habituated to find the food at the six focal trial sites during a two-week period before the trials started. The distance (30 cm) between the pile with food and the pile with novel items was also much greater than in our case where the teaspoon and the hen feathers were placed together with the quail eggs (figure 1). These differences may explain why the latency to start feeding was a matter of only a few minutes in their study versus a matter of hours and days in ours.

In the study in England, no difference was found between screws with a shiny silver colour versus screws painted blue with matte aerosol paint [5]. Thus, novel items do not need to be shiny to elicit a neophobic response in magpies, but shiny items may be more readily detected. In our trials, the magpies hardly moved the teaspoon, only the eggs, while many ravens removed both the eggs and the teaspoon. Avoidance of touching the novel items was also found in the magpie study in England. In the latter study, neophobia was only observed in free-living birds but not in captive magpies. The latter birds may have been more habituated to anthropogenic material.

A problem is that such nest decoration may increase the detectability of the nest by predators. Therefore, the benefit may depend heavily on circumstances, such as the amounts of solid wastes in the neighbourhood to which the potential nest predators have become habituated. Decorating the nest with anthropogenic material may have no or even a negative effect on some open-nesting birds, like in terns and gulls that often use such nest material [47]. In these birds, many nests are often built within a small area and are easily found. It should be investigated how soon nest predators habituate to the anthropogenic material used in the nests, like the ravens did at the landfill in the present study. Anthropogenic nest material may increase the visibility of a nest, not only by corvids but also by conspecifics [2]. For instance, in the clay-coloured thrush *Turdus grayi*, exposed anthropogenic material reduced nest survival in an urban area. The principal predators were conspecifics and not corvids [10]. A final case are birds where the nest is so well hidden that decoration with anthropogenic material would not increase detectability to nest predators. Artificial material may be found in nests of small, hole-nesting passerine birds but usually in small amounts [15,48].

### 4.2. The Fear of Feathers Hypothesis

Birds may add feathers to their nest for several reasons, primarily for insulation, but also for structural support and for status signalling [20]. The Fear of Feathers Hypothesis assumes that the presence of some large, reflective feathers may elicit a fear response in other birds and thus cause a reluctance to enter the nest and a reduction in the risk of nest usurpation, egg dumping and nest predation [36,38]. Previous experiments on cavity-nesting birds showed that rivals of the same or of another species, prospecting for a nest cavity, hesitated to enter an unfamiliar nest decorated with a few white feathers but not when decorated with similar-sized pieces of white paper, showing that it was the feathers themselves that caused the aversion and not the white objects [36]. White feathers can be seen by an intruder already from the entrance of a nest cavity before any damage to the nest content has been done.

In the present study, the Fear of Feathers Hypothesis was supported because nest predation by both species of corvids was delayed for nests decorated with large, white feathers compared to the depredation of the control nests. This was not obvious because a group of feathers may indicate the remains of a dead bird that scavengers such as corvids may visit when looking for food. According to a review of nest-building behaviour of birds that are present in the current part of Norway, at least 32 open- and 13 cavity-nesting species use feathers in their nest, in addition to swans, geese and ducks that all use downy feathers [49]. In the present study, the hen feathers were 3−13 cm long. Even small birds, such as blue tits, may decorate the nest with feathers that are up to 12.5 cm long (T Slagsvold, unpublished data).

Møller [37] offered artificial nests along lines with a distance of 100 m between each, and with three plasticine eggs in each second nest. The result was opposed to ours because nests with feathers were depredated sooner than nests without. However, breast feathers from a female pheasant *Phasianus colchinus* were added to each nest, feathers that are grey-brownish and much smaller than the white hen feathers that we used. We conclude that the effect of feathers on nest predation is strongly context dependent.

### 4.3. Mammalian versus avian nest predators

A problem in open-nesting birds is that although white, reflective feathers may delay nest predation by corvids, it may increase detectability by mammalian predators which often hunt in poor light conditions. Mammals tend to be less neophobic than birds [26] and they may not only cause a threat to eggs and nestlings but also to the adult birds themselves when incubating and brooding. In our study, egg removal by the red fox did not seem to be negatively affected by the presence of a teaspoon or white hen feathers in a nest. Mammalian predators, like foxes, show opportunistic feeding habits and can soon adapt to new environments and sources of food [50]. The foxes are attracted to forage at landfills where they soon may habituate to search for food among anthropogenic material. Increased detectability caused by nest decoration, combined with low levels of neophobia, may explain why the mammals did not target their first predation of a control nest as often as the corvids did. A difference between avian and mammalian nest predators may also result from their method of searching for food (by sight, hearing and odour) [32,51,52]. However, in the present study, the difference was probably of minor importance because the food provided (eggs) was of the same type and amount in each artificial nest and the nests were near each other.

### 4.4. Methods to study nest predation

When studying nest predation, important questions are the frequency of predation, which species of predators are involved, and how soon the predator approaches a nest after discovery. When conducting experiments on nest predation in the wild, a problem is that it may take a long time before the focal predator discovers the nests involved. Nests may be observed by a predator from a distance and thus it may be difficult to know the exact time of discovery and therefore to know the time elapsing from the first discovery of a nest to the depredation. In the study area, previous experiments have shown that concealed, artificial nests suffered low predation rate when placed on the ground [30]. Therefore, in the present study, all three nests of a trial were placed openly on the ground and contained exposed eggs. The nests were near each other and one nest was always decorated with white feathers, presumably causing all nests to be detected easily and simultaneously (figure 1).

We used fresh quail eggs and artificial nests. The method seems to simulate natural cases quite well [43,44]. However, our nests had no owner visiting or present at the nest that might increase detectability by searching predators, nor a parent to cover the eggs and nestlings and to attack approaching enemies [24]. It needs to be studied whether the corvids observe the behaviour of the nest owners from a distance to obtain information on the danger involved visiting a nest. For instance, if the owners enter the nest with no sign of fear it may stimulate corvid predation. Our study is somewhat unrealistic to many bird species because we presented three artificial nests simultaneously in close proximity. However, note that we focused most of our attention on the first nest to be depredated.

In our study area, previous experiments on predation of quail and plasticine eggs presented in open nests have shown that predation rates were similar for natural and artificial nests both in woodlands [53] and in open landscapes [53,54], although no parent bird was present at the artificial nests. However, the question remains whether the present results are relevant to natural, active nests. We used unoccupied nests because previous experiments have shown that if we add new objects, such as feathers to bird nests, they may soon be removed by the owners (T Slagsvold, unpublished data). If adding novel items, such as pieces of plastic or metal, there is also a risk that the owners may desert the nest. We recommend that birds that use anthropogenic material in their nest are selected for further tests of the Neophobia Hypothesis. Hopefully, the nest owners will accept both removal and addition of novel items after incubation has started.

In the present study, the first egg was most often removed from a control nest (figure 3) and in particular when a short time elapsed before depredation started. After such experience, the focal predator may have perceived the other, nearby nests as less dangerous. Therefore, our results of reluctance to remove an egg from a decorated nest may have been conservative. Note also that the nests were usually put out in the morning and mid-day. Ravens are quite shy birds and many of them avoided feeding at the landfill when people were working there. This probably caused nest predation to be delayed. Most predation occurred after the landfill was closed in the afternoon. The magpies were mostly studied in gardens, but people were often away during the daytime. Thus, the result of earlier depredation by ravens than by magpies was probably conservative because the magpies could spend more time observing the nests without being disturbed by humans.

## 5. Conclusion

Corvids are not only among the most serious predators on birds’ nests in nature but also among the most neophobic of any bird. When we provided quail eggs in artificial nests in the wild, both magpies and ravens often spent a long time before starting to remove eggs. Both species were less likely to remove eggs from nests decorated with a shiny metal teaspoon, or with large, white hen feathers. Thus, the study supports the idea that birds may suffer less nest predation by corvids if they decorate the nest with such material. However, the decoration may increase the attention by nest predators. In our study at the landfill, red foxes did not avoid the decorated nests.

In contrast to feathers, anthropogenic materials, such as plastic, have not been around much until recently which is reflected in the more frequent use in birds´ nests in recent years [55]. Further experiments are needed to study to which extent this increase in use is not only a result of increased availability but also affected by habituation to artificial material, to a spread of the behaviour through social learning and/or by natural selection through increased reproductive success. In birds, social learning may have some importance for the choice of nest material [56], perhaps mainly through ´horizontal transmission´ (e.g. colour of wool used as lining material in tits) [57]. However, in most birds, choice of nest material seems to have a strong genetic basis. For example, blue tits decorate the nest with many feathers whereas the great tits *Parus major* uses few feathers [28]. Cross-fostering showed that the difference between the two species was maintained when birds were raised by the other species [28]. Perhaps cultural transmission is more relevant to some types of anthropogenic material that persist in the nest and is exposed for learning for a longer period than feathers [6].

## Data Availability

The data is available as supplementary material [58].
